# Genetic dissection for head blast resistance in wheat using two mapping populations

**DOI:** 10.1038/s41437-021-00480-3

**Published:** 2021-12-08

**Authors:** Xinyao He, Muhammad Rezaul Kabir, Krishna K. Roy, Felix Marza, Aakash Chawade, Etienne Duveiller, Carolina Saint Pierre, Pawan K. Singh

**Affiliations:** 1 International Maize and Wheat Improvement Center (CIMMYT), Mexico, DF Mexico; 2 Bangladesh Wheat and Maize Research Institute (BWMRI), Dinajpur, Bangladesh; 3 Instituto Nacional de Innovación Agropecuaria y Forestal (INIAF), La Paz, Bolivia; 4 Department of Plant Breeding, Swedish University of Agricultural Sciences, Alnarp, Sweden

**Keywords:** Biotic, Plant breeding

## Abstract

Wheat head blast is a dangerous fungal disease in South America and has recently spread to Bangladesh and Zambia, threatening wheat production in those regions. Host resistance as an economical and environment-friendly management strategy has been heavily relied on, and understanding the resistance loci in the wheat genome is very helpful to resistance breeding. In the current study, two recombinant inbred line (RIL) populations, Alondra/Milan (with 296 RILs) and Caninde#2/Milan-S (with 254 RILs and Milan-S being a susceptible variant of Milan), were used for mapping QTL associated with head blast resistance in field experiments. Phenotyping was conducted in Quirusillas and Okinawa, Bolivia, and in Jashore, Bangladesh, during the 2017–18 and 2018–19 cropping cycles. The DArTseq^®^ technology was employed to genotype the lines, along with four STS markers in the 2NS region. A QTL with consistent major effects was mapped on the 2NS/2AS translocation region in both populations, explaining phenotypic variation from 16.7 to 79.4% across experiments. Additional QTL were detected on chromosomes 2DL, 7AL, and 7DS in the Alondra/Milan population, and 2BS, 4AL, 5AS, 5DL, 7AS, and 7AL in the Caninde#2/Milan-S population, all showing phenotypic effects <10%. The results corroborated the important role of the 2NS/2AS translocation on WB resistance and identified a few novel QTL for possible deployment in wheat breeding. The low phenotypic effects of the non-2NS QTL warrantee further investigation for novel QTL with higher and more stable effects against WB, to alleviate the heavy reliance on 2NS-based resistance.

## Introduction

Wheat blast (WB) is an emerging fungal disease with great potentiality in causing large-scale yield reduction in the tropical and subtropical wheat production areas. The disease is caused by the ascomycete fungus *Magnaporthe oryzae* pathotype *Triticum* (MoT) under warm and humid conditions (Cruz and Valent 2017; Kohli et al. 2011). Traditional epidemic regions include South American countries Brazil, Bolivia, Paraguay, and Argentina, and in the last few years, WB incidence has been reported in Bangladesh (Malaker et al. 2016) and Zambia (Tembo et al. 2020), posing a great threat to their neighboring countries in Asia and Africa, respectively, considering the fact that MoT is both seed- and air-borne. Based on climate analogy, WB vulnerable areas of 7 million ha were identified in India, Pakistan and Bangladesh, from where an annual yield reduction of 0.89–1.77 million tons of wheat may occur under WB conducive conditions (Mottaleb et al. 2018). Additional WB-prone areas have been identified in the USA, Ethiopia and Australia, especially under the scenario of global warming (Cao et al. 2011; Cruz et al. 2016a; Duveiller et al. 2011; Maciel 2011).

Although MoT can infect all the aerial parts of wheat, head blast on spikes is the most conspicuous symptom, often causing severe yield losses under favorable conditions on susceptible wheat varieties (Kohli et al. 2011). Disease development of WB is very fast, and the entire spikes may become bleached only a few days after the first symptom is seen, leaving no time for remedial actions like fungicide application (Duveiller et al. 2016). In South America, farmers often apply fungicides two to three times around the anthesis period to prevent this disease, yet severe yield loss may occur, as demonstrated in Brazil during the 2005 WB epidemic, when 14–32% yield reduction was recorded for two widely grown varieties despite two applications of fungicide (Urashima et al. 2009). In addition to the low effectiveness, the fast development of resistant fungal isolates is another factor to compromise fungicidal application, due to the high evolving rate of MoT (Castroagudín et al. 2015). This is also a major difficulty for resistance breeding, and there have been many varieties initially identified as WB resistant that became susceptible in later experiments or in large-scale cultivation (Cruz and Valent 2017). Nevertheless, varietal resistance has been heavily relied on both in South America and South Asia, due to its low cost and ease of adoption, which is especially important for small-scale, resource-poor farmers having limited access to other WB management tools.

Great efforts have been made on germplasm screening for WB resistance, yet no immunity has been identified, and the source of resistance has been limited, mostly being 2NS/2AS translocation carriers (Cruppe et al. 2020a; He et al. 2021; Juliana et al. 2020). This translocation was introduced from *Aegilops ventricosa* to utilize the rust resistance genes *Lr37*, *Sr38*, and *Yr17* (Helguera et al. 2003) and was later found to confer good WB resistance (Cruz et al. 2016b). Although its effect on WB resistance has been eroded by new MoT isolates (Cruppe et al. 2020a), 2NS-based resistance remains to be the corner-stone of the control strategy in most WB epidemic regions, where the large-scale sown varieties are mostly 2NS carriers, such as Sossego and CD 116 in Brazil, Caninde#1 in Paraguay, and Urubo and INIAF Tropical in Bolivia (Cruppe et al. 2020a; He et al. 2021). Due to the significant effects on yield advantage and rust resistance, the frequency of genotypes harboring 2NS has increased to around 90% in CIMMYT materials released after 2015 (Juliana et al. 2020; Juliana et al. 2019).

Genetics for host resistance against WB has mostly been investigated at the seedling stage, and a few resistance genes have been identified, including *Rmg2*, *Rmg3*, *Rmg7*, *Rmg8*, and *RmgGR119*, of which the latter three also conferred resistance against head blast infection (He et al. 2020a; Singh et al. 2021). According to Cruz and Valent (2017), however, *Rmg7* has been defeated by recent MoT isolates, whereas *Rmg8* and *RmgGR119* still need to be tested against the new MoT isolates in field experiments. Recent genetic studies on head blast resistance have confirmed the major effects of 2NS/2AS translocation, along with a few QTL or marker-trait associations (MTA) with minor effects that were less stably expressed across environments or experiments (Cruppe et al. 2020b; Ferreira et al. 2020; Goddard et al. 2020; He et al. 2021; Juliana et al. 2020; Wu et al. 2021).

In our previous study on the Caninde#1/Alondra population, a major QTL for head blast resistance was identified on the 2NS/2AS translocation region, explaining phenotypic effects between 22.4 and 50.1% across 12 field experiments, and minor QTL were identified on chromosomes 1AS, 2BL, 3AL, 4BS, 4DL, and 7BS (He et al. 2020b). The aims of the present study were to map two more populations, Alondra/Milan and Caninde#2/Milan-S, for head blast resistance and to identify molecular markers suitable for marker-assisted selection (MAS).

## Materials and methods

### Plant material

Two recombinant inbred line (RIL) populations, Alondra/Milan (referred to as AM hereafter) with 296 RILs and Caninde#2/Milan-S (CM) with 254 RILs, were developed via the single seed descend method to F_2:7_ generation. The parent Alondra has a pedigree of D-6301/NAINARI-60//WEIQUE/RED-MACE/3/CIANO-F-67*2/CHRIS, which is a 2AS carrier and is susceptible to WB. Caninde#2 is a 2NS variety released in Paraguay due to its good WB resistance (Duveiller et al. 2016), with a pedigree of ITAPUA-35-APEREA/PF-84432//CORDILLERA-4. The parent Milan is a well-known CIMMYT wheat line that has a pedigree of VS-73-600/MAIORAL/3/BOBWHITE/YECORA-70//TRIFON. It has frequently been used as a WB resistance donor and many of its derivatives also have good WB resistance due to the presence of the 2NS/2AS translocation (Kohli et al. 2011). However, in our recent study, residual heterozygosity was found in the 2NS/2AS translocation region of Milan, and the presence of WB susceptible Milan lines (denoted as Milan-S) that have the 2AS segment were also reported (He et al. 2021). The Caninde#2/Milan-S population was initially made for breeding purposes, but it demonstrated strong segregation of WB resistance among the RILs, implying that Milan-S, instead of a 2NS Milan line (Milan-R), was used to generate the population. Therefore, the Caninde#2/Milan-S population was also included in this study to map WB resistance.

### Inoculum preparation

The inoculum used in this study was composed of locally collected MoT isolates that had shown a high capacity of sporulation, including OKI1503, OKI1704, QUI1505, QUI1601, and QUI1612 in Bolivia and BHO17001, MEH17003, GOP17001.2, RAJ17001, CHU16001.3, and JES16001 in Bangladesh. The isolates were grown on oatmeal agar plates for sporulation, and the harvested conidia were adjusted to a concentration of 80,000 spores/ml using a hemocytometer under a microscope, and finally, Tween-20 was added to make a concentration of 0.02% before field application. For more technical details, refer to He et al. (2020b).

### Field experiments

The field experiments were conducted in three locations over 2 years, involving the 2017–18 and 2018–19 cycles in Quirusillas (Bolivia) and Jashore (Bangladesh), and the 2018 and 2019 cycles in Okinawa (Bolivia). Two sowings that were separated by around 2 weeks were made in each location to expose the populations to wider environmental conditions, and no replication was made within each sowing. The experiments in this study were denoted according to location (“Quir” for Quirusillas, “Jash” for Jashore, and “Oki” for Okinawa), cropping cycle (“18” for the 2017–18 or 2018 cycle, and “19” for the 2018–19 or 2019 cycle), and sowing (“a” and “b” for the first and second sowings, respectively). Both Quirusillas and Okinawa are located in the Department of Santa Cruz of Bolivia, but the former is in the high land region with a cropping season from December to April, and the latter is in the lowland region with a cropping season from May to August. Jashore is located in the Division of Khulna, Bangladesh, and has a cropping cycle from December to April.

The two mapping populations were sown in 1 m double rows spaced 20 cm apart. To create a WB conducive micro-environment, a misting system was used in the field that worked 10 min each hour from 8 a.m. to 7 p.m. The resistant checks used were Urubo in Bolivia and BARI Gom 33 in Bangladesh, and the corresponding susceptible checks were Atlax and BARI Gom 26, respectively. At the anthesis stage, ten spikes were tagged with sticky paper tape for later WB evaluation, which were selected from different plants of a RIL to increase representativity, and the spikes were spray inoculated with a CO_2_ driven backpack sprayer in the evening. Inoculation was repeated 2 days later to ensure that late spikes were inoculated. According to the disease progress, field evaluation was conducted at 14 or 21 days after the first inoculation, and the ten inoculated and marked spikes were counted for numbers of total spikelets and that of infected spikelets. WB index was calculated via multiplying *Incidence* and *Severity*, with the former being the percentage of infected spikes and the latter the averaged percentage of infected spikelets. Additionally, days to heading (DH) and plant height (PH) were evaluated in all the experiments to investigate their potential association with WB resistance.

### Statistical analysis

The PROC GLM module of SAS ver. 9.2 was used to perform analysis of variance (ANOVA), and the PROC CORR module was employed to calculate Pearson correlation coefficients. Broad sense heritability estimates were calculated with the formula *H*^*2*^ = $\sigma _g^2$/($\sigma _g^2$ + $\sigma _{g \,\ast\, y}^2$/*y* + $\sigma _{g \,\ast\, s}^2$/*s* + $\sigma _e^2$/*sy*), where $\sigma _g^2$ is for genetic variance, $\sigma _{g \,\ast\, y}^2$ for genotype-by-year interaction, $\sigma _{g \,\ast\, s}^2$ for genotype-by-sowing interaction, $\sigma _e^2$ for error variance, *y* for the number of years, and *s* for the number of sowings.

### Genotyping

Genomic DNA was isolated from young leaves with the CTAB method and then was subjected to the DArTseq^®^ genotyping platform at the Genetic Analysis Service for Agriculture (SAGA) at CIMMYT, Mexico. Additionally, four STS markers in the 2NS/2AS region were assayed in this study, including *Ventriup-LN2* (Helguera et al. 2003), *WGGB156* and *WGGB159* (Wang et al. 2018), and *cslVrgal3* that was developed from a follow-up study of Seah et al. (2001) (E. Lagudah, pers. comm.). Marker screening was conducted with the thresholds of 20% missing data points and 30% of minor allele frequency. Redundant markers were excluded from further analysis with the BIN module of the ICIMapping v. 4.1 software (www.isbreeding.net).

### Linkage and QTL mapping

Linkage analysis was carried out with the JoinMap v.4 software (Van Ooijen 2006), using LOD scores from 5 to 10 for grouping individual linkage groups (LG) and the Maximum Likelihood algorithm for ordering markers in each LG. Assignment of LGs to chromosomes was done via BLAST searches of the DArTseq marker sequences against the Chinese Spring genome (CS IWGSC RefSeq v1.0). QTL analysis followed a two-step procedure using MapQTL v6.0 (Van Ooijen 2009), in which interval mapping (IM) was first used to detect potential QTL and their tightly linked markers. Then multiple QTL mapping was carried out for each QTL, using its most closely linked marker identified in IM as a cofactor. Significant QTL were reported when they have a LOD score of >3.0 in at least one experiment or >2.0 in multiple experiments. MapChart ver. 2.3 (Voorrips 2002) was used to draw LGs and LOD profiles.

## Results

### Phenotyping

**Fig. 1:**
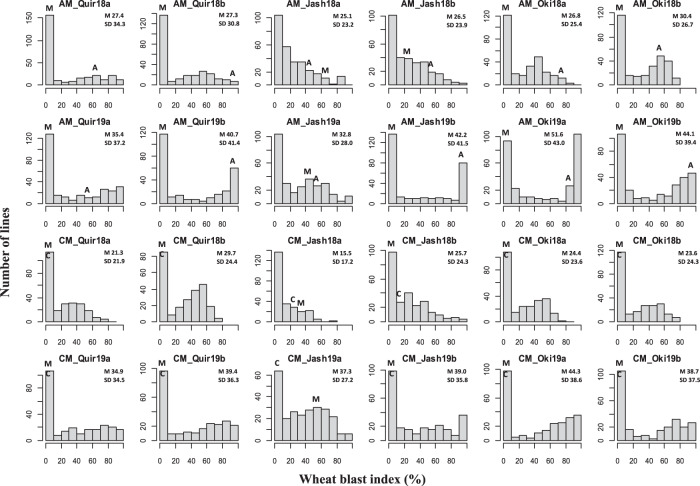
Histograms of wheat blast index for the populations Alondra/Milan (AM) and Caninde#2/Milan-S (CM) in individual experiments. Phenotypic ranges of the two parents are indicated, where A stands for Alondra, C for Caninde#2 and M for Milan-S. “Quir” stands for Quirusillas, “Jash” for Jashore, and “Oki” for Okinawa, “18” and “19” for the 2017–18 or 2018 cycle and 2018–19 or 2019 cycle, respectively, and “a” and “b” for the first and second sowing, respectively. Grand mean (M) and standard deviation (SD) values are presented for all experiments.

Disease pressure varied greatly across the 12 experiments. Jash18a exhibited the lowest grand means of WB index for the two populations, i.e., 25.1% for AM and 15.5% for CM, and Oki19a showed the highest infection with 51.6% for AM and 44.3% for CM (Fig. 1). In AM, the resistant parent Milan significantly outperformed the susceptible parent Alondra in most experiments, whereas both parents appeared to be similarly resistant in CM, due to the use of Milan-R instead of Milan-S. Another observation is that Milan performed better in Quirusillas and Okinawa than in Jashore, possibly due to genotype-by-environment interaction (Fig. 1). ANOVA results indicated significant effects of “Genotype” and “Genotype × Year” in both populations across all locations, and moderately high heritability estimates ranging from 0.71 to 0.88 for AM and from 0.73 to 0.86 for CM were obtained (Table 1). Significant correlations of WB index were found among all experiments, with *r*-values ranging from 0.30 to 0.90 for AM and 0.32 to 0.85 for CM. Generally, experiments in Bolivia exhibited a better correlation than those in Bangladesh (Table 2). Correlation of WB with DH and PH was mostly nonsignificant, and the few significant correlations were of low levels (Table S1). Between WB and PH, the significant correlations were all negative, except for Jash19b, in agreement with the general trend that tall plants have lower WB. Between WB and DH, all significant correlations in Jashore were positive, whereas those in Quirusillas and Okinawa were negative (Table S1).

**Table 1:** Analysis of variance for wheat blast index and its heritability estimates in different locations for the populations Alondra/Milan (AM) and Caninde#2/Milan-S (CM).

Pop.	Location	Source	DF	Mean square	*F* value	*P* value	Heritability
AM	Quirusillas	Genotype	293	3354.35	10.76	<0.0001	0.71
		Year	1	40187.86	128.96	<0.0001	
		Sowing (year)	1	2423.97	7.78	0.0057	
		Genotype × year	275	998.90	3.21	<0.0001	
		Genotype × sowing	287	305.51	0.98	0.5652	
		Error	246	311.62			
	Jashore	Genotype	295	2175.23	5.19	<0.0001	0.72
		Year	1	39543.05	94.37	<0.0001	
		Sowing (year)	1	4444.87	10.61	0.0013	
		Genotype × year	295	587.40	1.40	0.0019	
		Genotype × sowing	295	437.37	1.04	0.3567	
		Error	293	419.01			
	Okinawa	Genotype	295	3917.95	24.59	<0.0001	0.88
		Year	1	109617.20	687.98	<0.0001	
		Sowing (year)	1	8365.98	52.51	<0.0001	
		Genotype × year	294	467.55	2.93	<0.0001	
		Genotype × sowing	295	162.81	1.02	0.4273	
		Error	287	159.33			
CM	Quirusillas	Genotype	253	2510.31	12.60	<0.0001	0.75
		Year	1	32988.66	165.58	<0.0001	
		Sowing (year)	1	1009.74	5.07	0.0253	
		Genotype × year	252	614.32	3.08	<0.0001	
		Genotype × sowing	250	212.20	1.07	0.3112	
		Error	241	199.23			
	Jashore	Genotype	253	1714.67	4.64	<0.0001	0.73
		Year	1	78005.70	210.99	<0.0001	
		Sowing (year)	1	4441.59	12.01	0.0006	
		Genotype × year	253	469.93	1.27	0.0285	
		Genotype × sowing	253	367.98	1.00	0.5149	
		Error	253	369.72			
	Okinawa	Genotype	253	3167.07	13.96	<0.0001	0.86
		Year	1	78443.71	345.74	<0.0001	
		Sowing (year)	1	1353.29	5.96	0.0153	
		Genotype × year	253	443.17	1.95	<0.0001	
		Genotype × sowing	253	219.16	0.97	0.6084	
		Error	252	226.89

**Table 2:**
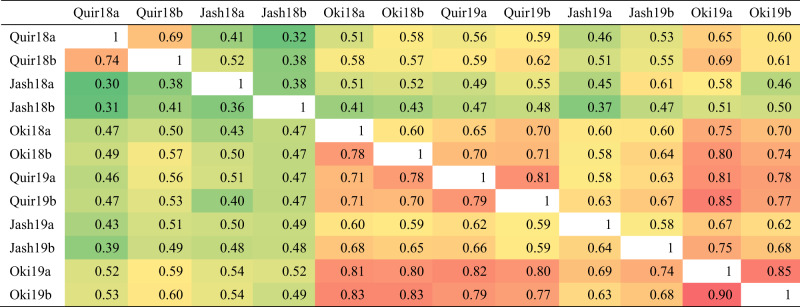
Pearson correlation coefficients of wheat blast index across experiments for the populations Alondra/Milan (below the diagonal) and Caninde#2/Milan-S (above the diagonal).

All correlations were significant at *P* < 0.0001. “Quir” stands for Quirusillas, “Jash” for Jashore, and “Oki” for Okinawa, “18” and “19” for the 2017–18 or 2018 cycle and 2018–19 or 2019 cycle, respectively, and “a” and “b” for the first and second sowing, respectively. Cell shades change from green to red with the increase of correlation coefficients.

### Genotyping and linkage analysis

After marker screening, 1452 and 1445 high-quality non-redundant markers were respectively obtained for AM and CM for linkage analysis, generating 39 LGs for the former and 40 LGs for the latter population. These LGs represent all 21 wheat chromosomes, of which 2B had the highest number of markers (169) and 4D had the lowest number of markers (18) for AM, and the corresponding chromosomes for CM are 5B (152) and 4D (7). These LGs covered a total genetic distance of 4979 cM with an average distance between markers of 3.4 cM for AM, and 3370 cM with 2.3 cM for CM (Tables S2, S3).

### QTL mapping

**Fig. 2:**
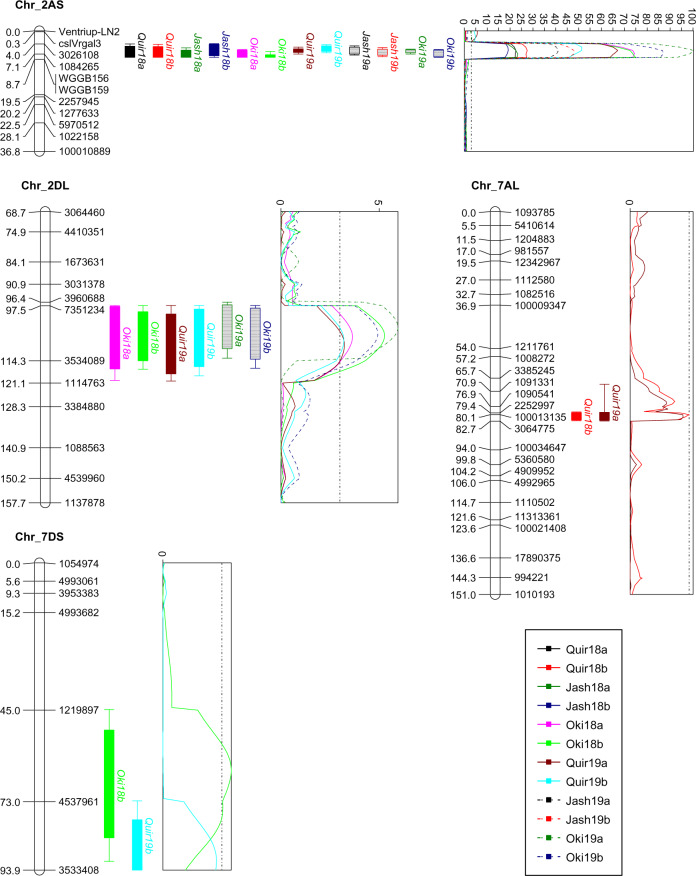
QTL profiles for wheat blast resistance in the Alondra/Milan population across experiments. Genetic distances are shown in centimorgans to the left side of the linkage groups. Only framework markers are presented except for the QTL region where all markers are shown. A threshold of 3.0 is indicated by a dashed vertical line in the LOD graph.

In the AM population, four QTL were detected on chromosomes 2AS, 2DL, 7AL, and 7DS, of which only the first one on 2NS/2AS was consistently significant across experiments, explaining phenotypic variation (PV) from 26.0 to 79.4%, whereas other QTL were of minor effects and were less consistent across experiments (Table 3 and Fig. 2). The 2DL QTL was significant in six out of 12 experiments, being the second most repeatable QTL and explaining PV from 4.9 to 9.0%. The 7AL and 7DS QTL were both significant in only two experiments. Milan contributed the resistant allele of the QTL on 2NS/2AS, whereas the susceptible parent Alondra contributed those of the remaining three QTL (Table 3).

**Table 3:** Phenotypic effects (%) of QTL for wheat blast index across 12 environments for the populations Alondra/Milan (AM) and Caninde#2/Milan-S (CM).

Pop.	Chr.	Position	Left marker	Right marker	Quir18a^a^	Quir18b	Jash18a	Jash18b	Oki18a	Oki18b	Quir19a	Quir19b	Jash19a	Jash19b	Oki19a	Oki19b	R source^b^
AM	2AS	4.0–8.7	3026108	WGGB156	30.8	36.9	30.5	26.0	68.6	68.8	66.5	59.1	47.3	52.6	79.4	74.8	M
	2DL	96.4–121.1	3960688	1114763					5.3	7.6	4.9	5.1			9.0	7.5	A
	7AL	79.4–82.7	2252997	3064775		4.7					4.1						A
	7DS	45.0–93.9	1219897	3533408						5.0		4.4					A
	Accumulated percentage of variation explained				30.8	41.6	30.5	26.0	73.9	81.4	75.5	68.6	47.3	52.6	88.4	82.3	
CM	2AS	0.0–6.6	3026108	1380019	36.9	39.9	25.8	16.7	48.7	45.2	52.5	59.0	44.6	42.2	68.9	59.5	C
	2BS	119.3–121.0	5970456	100007209			9.9							6.4			M
	4AL	49.6–53.9	2257480	1054303									4.4	4.1			C
	5AS	0.0–9.3	1095048	3064620							4.2	3.8			5.3	4.4	C
	5DL	49.0–53.5	1276750	4021694	5.8		4.7	4.3	4.4	4.4	3.4	3.7		4.1	6.0	5.6	C
	7AS	88.6–89.5	1063507	1062849							4.5	4.9				6.4	C
	7AL	40.0–43.5	1091331	1695112					5.6		4.2	5.1		3.5	4.4		C
	Accumulated percentage of variation explained				42.7	39.9	40.4	21.0	58.7	49.6	68.8	76.5	49.0	60.3	84.6	75.9	

^a^The percentages of phenotypic variation explained are shown in the table. “Quir” stands for Quirusillas, “Jash” for Jashore, and “Oki” for Okinawa, “18” and “19” for the 2017–18 or 2018 cycle and 2018–19 or 2019 cycle, respectively, and “a” and “b” for the first and second sowing, respectively.

^b^Source of resistance, where ***A*** for Alondra, ***C*** for Caninde#2, and ***M*** for Milan-R (2NS carrier) in AM and Milan-S (2AS carrier) in CM.

**Fig. 3:**
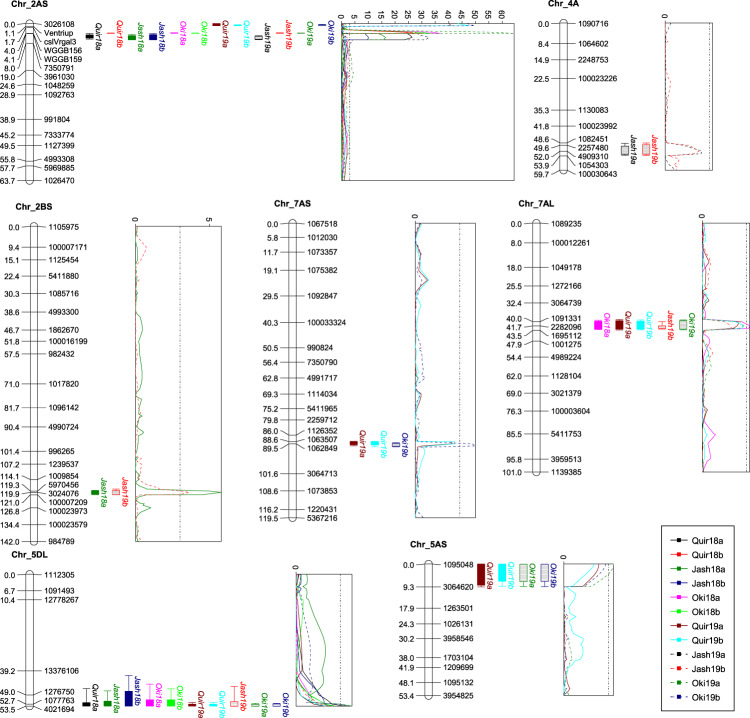
QTL profiles for wheat blast resistance in the Caninde#2/Milan-S population across experiments. Genetic distances are shown in centimorgans to the left side of the linkage groups. Only framework markers are presented except for the QTL region where all markers are shown. A threshold of 3.0 is indicated by a dashed vertical line in the LOD graph.

In the CM population, seven QTL were identified on chromosomes 2AS, 2BS, 4AL, 5AS, 5DL, 7AS, and 7AL. Again the QTL on 2NS/2AS was the only stably expressed QTL in all experiments, accounting for PV from 16.7 to 68.9%, and the rest QTL were all of minor effects with PV less than 10% (Table 3 and Fig. 3). The 5DL QTL was significant in 10 out of the 12 experiments, explaining PV from 3.4 to 6.0%, and the rest of QTL were significant in only two to five experiments. The resistant parent Caninde#2 contributed most resistance alleles, and Milan-S contributed only one for the 2BS QTL (Table 3).

Obviously, the 2AS QTL in both populations is associated with the 2NS/2AS translocation region, which is on the distal region of 2AS that corresponds to a physical range of 4.0–21.2 Mb in the CS genome, delimited by DArTSeq markers *3026108* and *1380019* (Tables 3, S4, Figs. 2, 3). The four STS markers *Ventriup-LN2*, *cslVrgal3*, *WGGB156*, and *WGGB159* were found in this region, with the first two mostly distal to and the last two mostly proximal to the WB QTL (Figs. 2, 3). Additionally, QTL on 7AL in the two populations might be the same, based on a comparison of the genetic (Fig. S1) and physical positions (675.4–685.1 Mb) of the flanking markers.

QTL mapping for DH (Table S5) and PH (Table S6) were also conducted to identify potential loci associated with WB resistance. There was no such locus identified in the AM population, and only one DH QTL on 4AL identified in the CM population showed linkage with WB QTL (Tables 3, S5), in agreement with the low correlation between WB resistance and DH/PH (Table S1).

## Discussion

Due to the fast-spreading of WB into new continents and its devastating nature, researchers and breeders in many WB affected and threatened countries enhanced or initiated genetic studies on WB resistance in the hope to identify major QTL and their linked molecular markers that could be used in MAS. As far as we know, seven of such studies have been published, of which six reported the 2NS/2AS translocation as the only stably expressed QTL with major effects (Cruppe et al. 2020b; Ferreira et al. 2020; He et al. 2021; He et al. 2020b; Juliana et al. 2020; Wu et al. 2021). The remaining one by Goddard et al. (2020) targeted a non-2NS source BR 18-Terena with moderate WB resistance and identified several non-2NS resistance loci at seedling and head stages via in vitro experiments. Although a few QTL appeared to have PVs higher than 10%, a conventional threshold for major QTL, their effects need to be confirmed in field experiments before being used in breeding. In the current study, the 2NS/2AS region appeared to be the only consistent QTL in both the AM and CM populations, whereas none of the rest QTL was consistently identified across experiments, again demonstrating the narrow genetic base for WB resistance.

It is widely acknowledged that alien chromosome segments do not recombine with their homoeologous wheat chromosome regions, which is especially true for those alien fragments introduced via *ph1b* mutant or irradiation technologies (Qi et al. 2007). However, due to the close evolutional relationship between wheat and *Aegilops* species, spontaneous translocation often happens between genomes of the two species (Doussinault et al. 1983). Furthermore, the introduced *Aegilops* chromosome segments are able to recombine at lower frequency with their counterparts in the wheat genome, which has been observed in the 2NS/2AS translocation region by many researchers (Bariana and McIntosh 1993; Bonhomme et al. 1995; Jahier et al. 2001; Robert et al. 2000). Recent QTL mapping studies where the 2NS/2AS translocation was involved also demonstrated the recombination within this region (Ferreira et al. 2020; He et al. 2020b; Xue et al. 2018). This enables the precision mapping (if not fine mapping) of the WB resistance QTL on the 2NS region.

Similar to our previous results in the Caninde#1/Alondra population (He et al. 2020b), the WB QTL in the 2NS region was mapped proximal to STS markers *Ventriup-LN2* and *cslVrgal3* and distal to *WGGB156* and *WGGB159*, except for three experiments for the CM population where the QTL was mapped distal to *Ventriup-LN2* and *cslVrgal3* (Fig. 3). By scrutinizing the LOD profiles, the peak region of this QTL appears more close to the STS marker *WGGB156* with a physical position in CS at 16.6 Mb. This is in agreement with the published results, where markers with the highest LOD (for bi-parental populations) or the lowest *p* value (for GWAS) were *S2A_16617246* at 16.6 Mb (Cruppe et al. 2020b), *AX-94469326* at 16.5 Mb (Ferreira et al. 2020), the DArTSeq marker *3024467* at 16.3 Mb (He et al. 2020b), *2A_18495181*, *2A_15449240*, *2A_14418760*, and *2A_18468495* ranging from 14.4 to 18.5 Mb in four GWAS panels (Juliana et al. 2020), *RAC875_c829_1215* at 17.0 Mb (He et al. 2021), and the DArTSeq marker *5323683* at 14.3 Mb (Wu et al. 2021). A recent study by Gao et al. (2021) estimated the size of the 2NS region in the US winter wheat variety Jagger to be 32.53 Mb, corresponding to a 24.64-Mb segment on the tip of 2AS in CS. Therefore, the WB QTL is likely to be at the proximal half of the 2NS segment, in which the codominant marker *WGGB156* is located, being a good candidate marker for diagnosing 2NS. Nevertheless, this marker has a drawback that the resistance allele with a 457-bp PCR product is associated with WB susceptibility in some Indian materials (He et al. 2021). Therefore, it is advisable to apply both *Ventriup-LN2* (or *cslVrgal3*) and *WGGB156* (or *WGGB159*) to improve the 2NS prediction. However, for breeding populations where the parental genotypes of *WGGB156* are determined, this codominant marker is advantageous since it could differentiate heterozygous genotype (2NS/2AS) from homozygous genotype (2NS/2NS), while preventing false negative results caused by poor DNA quality.

Although multiple minor QTL have been identified in the current study, few appeared to be mapped in previously published QTL regions. The QTL on chromosome 4AL in the CM population (physical range 479.0–538.9 Mb) is close to the one reported by Juliana et al. (2020) at 572.2 Mb, and they could represent the same QTL. Others, even though on the same chromosome, must be different QTL according to their physical positions, e.g., Goddard et al. (2020) reported a QTL on chromosome 2B, *QHead.jic-2B*, which must not be the same QTL as we found on 2BS in the CM population, due to the former being located on the 2BL chrmosome. Of all the minor QTL identified in the two populations, only one out of the nine QTL was contributed by Milan, probably implying a lack of minor WB resistance QTL in this genotype. Considering also the existence of Milan-S, it is more advantageous to utilize other 2NS donors like Caninde#1 and Caninde#2 in breeding programs for WB resistance, because the latters have several non-2NS QTL in addition to the major one on 2NS.

The experiments of the current study were conducted in parallel to those for the Caninde#1/Alondra population (He et al. 2020b), and similar results were obtained. Nevertheless, novelties of the current study can be identified in the identification of several non-2NS QTL that are mostly new, and characterization of the widely utilized CIMMYT line Milan in terms of its genetics for WB resistance. Additionally, this study confirmed the location of the 2NS/2AS QTL in two more bi-parental populations, and identified several DArTSeq markers flanking the WB QTL (Table S4). Although such markers cannot be directly used in MAS, they could be converted into KASP markers based on the sequence information, which has been done successfully in other studies (Gao et al. 2015).

In agreement with our previous reports (He et al. 2021; He et al. 2020b), DH and PH did not show a close association with field WB resistance in both AM and CM populations, which is in sharp contrast to Fusarium head blight, another major spike disease of wheat (Xu et al. 2020). The main reason could be the low variation in DH and PH among lines of these populations, which significantly reduced the difference in micro-environment among spikes of different lines. Another possibility could be a lack of tight linkage between QTL for DH/PH and those for WB resistance, leading to lower confounding effects of the two phenological traits on WB.

As an economical and easily adoptable management method, varietal resistance has been widely utilized in WB epidemic regions; however, most released cultivars with WB resistance are 2NS-based (Singh et al. 2021), making the resistance vulnerable to the fast evolving MoT isolates. Indeed, the 2NS-virulent strains have been identified in South America with increased frequency over time (Ceresini et al. 2018), making it imperative to search and utilize alternative WB resistance genes/QTL. Once the new resistant sources are identified, they will be utilized together or in rotation with the 2NS-based sources to reduce the high directional selection pressure that 2NS genotypes are imposing on the MoT population.

## Conclusion

The QTL on the 2NS/2AS translocation was the only major QTL identified in the two mapping populations, which is not cosegregating with the commonly utilized diagnostic marker *Ventriup-LN2*, warranting the utilization of additional markers in this regain to better diagnose the presence of 2NS. The multiple minor QTL could be potentially useful for WB resistance breeding if confirmed in other studies, in that case MAS is preferred considering their low phenotypic effects. Further efforts are imperative to identify non-2NS resistance with strong phenotypic effects for WB resistance breeding to slow down the evolution of MoT-virulent isolates.

## Supplementary information

Supplementary Material

## Data Availability

The raw genotypic and phenotypic data of this article are available at https://hdl.handle.net/11529/10548581.
