# Supplementary material for: Genetic dissection for head blast resistance in wheat using two mapping populations
Source: Heredity (Edinb). 2021 Dec 8;128(6):402–10. doi: 10.1038/s41437-021-00480-3 (PMC9177698; doi:10.1038/s41437-021-00480-3)
Supplement: Supplementary file 1 — Supplementary Material [file 41437_2021_480_MOESM1_ESM.docx]

**Table S1** Phenotypic correlation of wheat blast index with days to heading (DH) and plant height (PH) for the populations Alondra/Milan (AM) and Caninde#2/Milan-S (CM).

| **Pop.** | **Trait** | **Quir18a** | **Quir18b** | **Jash18a** | **Jash18b** | **Oki18a** | **Oki18b** | **Quir19a** | **Quir19b** | **Jash19a** | **Jash19b** | **Oki19a** | **Oki19b** |
| --- | --- | --- | --- | --- | --- | --- | --- | --- | --- | --- | --- | --- | --- |
| **AM** | **DH** | -0.15 | -0.10 | 0.13 | 0.23** | -0.07 | -0.07 | -0.16* | -0.04 | 0.09 | 0.03 | -0.03 | -0.22** |
|  | **PH** | -0.01 | -0.02 | -0.21** | -0.18* | -0.21** | -0.03 | -0.25** | -0.40** | -0.21** | -0.14 | -0.31** | -0.28** |
| **CM** | **DH** | -0.12 | -0.04 | 0.27** | 0.17* | 0.08 | -0.08 | 0.03 | 0.03 | 0.03 | 0.10 | 0.03 | 0.01 |
|  | **PH** | 0.05 | 0.11 | 0.07 | 0.04 | 0.11 | 0.13 | -0.04 | -0.01 | 0.02 | 0.17* | 0.03 | -0.25** |

‘Quir’ stands for Quirusillas, ‘Jash’ for Jashore, and ‘Oki’ for Okinawa, ‘18’ and ‘19’ for the 2017-18 or 2018 cycle and 2018-19 or 2019 cycle, respectively, and ‘a’ and ‘b’ for the first and second sowing, respectively.

* *p*<0.01, ** *p*<0.001

**Table S2**. Marker and linkage map statistics for the Alondra/Milan population

| Chromosome | Number of markers | Genetic length (cM) | Average distance (cM) between markers |
| --- | --- | --- | --- |
| 1A | 106 | 245 | 2.3 |
| 1B | 78 | 228 | 3.0 |
| 1D | 63 | 124 | 2.0 |
| 2A | 56 | 214 | 3.9 |
| 2B | 169 | 416 | 2.5 |
| 2D | 52 | 218 | 4.3 |
| 3A | 70 | 267 | 3.9 |
| 3B | 57 | 135 | 2.4 |
| 3D | 42 | 299 | 7.3 |
| 4A | 56 | 253 | 4.6 |
| 4B | 39 | 162 | 4.3 |
| 4D | 18 | 124 | 7.3 |
| 5A | 85 | 322 | 3.8 |
| 5B | 89 | 326 | 3.7 |
| 5D | 56 | 232 | 4.2 |
| 6A | 36 | 103 | 2.9 |
| 6B | 79 | 229 | 2.9 |
| 6D | 27 | 211 | 8.1 |
| 7A | 95 | 303 | 3.2 |
| 7B | 130 | 315 | 2.4 |
| 7D | 49 | 253 | 5.3 |
| Whole Genome | 1452 | 4979 | 3.4 |

**Table S3.** Marker and linkage map statistics for the Caninde#2/Milan-S population

| Chromosome | Number of markers | Genetic length (cM) | Average distance (cM) between markers |
| --- | --- | --- | --- |
| 1A | 105 | 193 | 1.9 |
| 1B | 73 | 127 | 1.8 |
| 1D | 17 | 68 | 4.3 |
| 2A | 116 | 291 | 2.5 |
| 2B | 137 | 316 | 2.3 |
| 2D | 11 | 70 | 7.0 |
| 3A | 94 | 237 | 2.5 |
| 3B | 140 | 300 | 2.2 |
| 3D | 19 | 109 | 6.1 |
| 4A | 64 | 96 | 1.5 |
| 4B | 53 | 108 | 2.1 |
| 4D | 7 | 33 | 5.5 |
| 5A | 69 | 146 | 2.1 |
| 5B | 152 | 305 | 2.0 |
| 5D | 16 | 77 | 5.1 |
| 6A | 80 | 133 | 1.7 |
| 6B | 96 | 207 | 2.2 |
| 6D | 32 | 158 | 5.1 |
| 7A | 101 | 232 | 2.3 |
| 7B | 40 | 95 | 2.4 |
| 7D | 23 | 69 | 3.1 |
| Whole Genome | 1445 | 3370 | 2.3 |

**Table S4.** Sequence information of the DArTSeq markers in the 2NS/2AS WB QTL region.

| **Marker ID** | **Marker type^&^** | **Physical position^#^** | **Marker sequence** |
| --- | --- | --- | --- |
| 3026108 | SNP | 398221 | TGCAGGCCGAC[C/G]AGCACGGACTTTGTTCGGTTGTTACCGAGATCGGAAGAGCGGTTCAGCAGGAATGCC |
| 5358624 | PAV | 11153317 | TGCAGTGGCTGGCCGTGAAGCACACCCCGAGATCGGAAGAGCGGTTCAGCAGGAATGCCGAGACCGATC |
| 39684922 | SNP | 11695252 | TGCAGATATGGCAGTCTCCATGGCT[T/G]GGATTGATGAGGTAAGCTGTTTGGCCGAGATCGGAAGAGCGGT |
| 4538755 | PAV | 13563682 | TGCAGGGTAGCACATGATGCACGAGTGTCCGAGATCGGAAGAGCGGTTCAGCAGGAATGCCGAGACCGA |
| 7351148 | SNP | 17830641 | TGCAGTGGC[A/G]GGTCACGTCCGCTTTAAACCGAGATCGGAAGAGCGGTTCAGCAGGAATGCCGAGACCGA |
| 3958902 | SNP | 18468451 | TGCAGATCCAACG[T/C]GAGCAAGCCAGGTTTGCGAACGTCCTCACCGAGATCGGAAGAGCGGTTCAGCAGG |
| 1084265 | SNP | 21192626 | TGCAGTACACGGC[G/A]TACATATTCTTCGGCGACGCCATCAACAGCAAGCCTCTGGAGGCCCTTCTCCTCA |
| 1119713 | PAV | 23502408 | TGCAGTATCAGTTGCTGAGTAGGGAGGAGGAGGGGAATCAGCCGTTGTACGGGTCTGCGGCCGAAGAAC |
| 1380019 | PAV | 24485285 | TGCAGGGGCGATGCGGGGCATGGAGCTGAGGCGACAACAGCAGTGAGCGCCGTGGGTGGCAGCGGGCGG |

^&^ SNP for Single Nucleotide Polymorphism markers, and PAV for Presence-Absence Polymorphism markers.

^#^ Physical positions in the Chinese Spring reference genome ver. 1.0 are shown.

**Table S5.** Phenotypic effects (%) of QTL for days to heading in the three locations for the populations Alondra/Milan (AM) and Caninde#2/Milan-S (CM).

| **Pop.** | **Chr.** | **Position** | **Left marker** | **Right marker** | **Quirusillas** | **Jashore** | | **Okinawa** | | **Source of earliness** | |
| --- | --- | --- | --- | --- | --- | --- | --- | --- | --- | --- | --- |
| **AM** | 1AS | 2.4-7.0 | 3021960 | 3532756 | 5.0 |  | |  | | Alondra | |
|  | 2DL | 21.1-22.8 | 3533235 | 1103039 | 5.3 |  | |  | | Milan | |
|  | 4AL | 152.3-162.4 | 1066988 | 13142775 | | | 6.9 | |  | | Alondra |
|  | 5AL | 217.6-230.7 | 2259187 | 1714015 | | | 8.7 | |  | | Milan |
|  | 7BS | 22.9-38.5 | 4409503 | 1090158 | 11.8 | 17.3 | | 16.8 | | Alondra | |
|  | Accumulated percentage of variation explained | | | | 22.1 | 32.9 | | 16.8 | |  | |
| **CM** | 2BS | 115.3-126.8 | 6036484 | 100023973 | 5.1 | 7.5 | | 5.1 | | Milan | |
|  | 4AL | 41.8-48.3 | 100023992 | 4993706 |  |  | | 5.8 | | Milan | |
|  | 4BL | 15.4-16.8 | 2332740 | 1113711 |  | 6.2 | |  | | Milan | |
|  | 5BS | 3.7-8.6 | 1013369 | 1057285 | 6.3 |  | |  | | Milan | |
|  | 7AL | 0-10.5 | 1218013 | 994027 | 7.0 |  | |  | | Caninde#2 | |
|  | 7DS | 36.3-37.0 | 1091583 | 100008445 |  | 11.2 | | 6.3 | | Milan | |
|  | Accumulated percentage of variation explained | | | | 18.4 | 24.9 | | 17.2 | |  | |

**Table S6.** Phenotypic effects (%) of QTL for plant height in the three locations for the populations Alondra/Milan (AM) and Caninde#2/Milan-S (CM).

| **Pop.** | **Chr.** | **Position** | **Left marker** | **Right marker** | **Quirusillas** | **Jashore** | **Okinawa** | **Source of high stature** |
| --- | --- | --- | --- | --- | --- | --- | --- | --- |
| **AM** | 2AS | 20.2-28.1 | 1277633 | 1022158 |  |  | 5.8 | Milan |
|  | 3AS | 78.6-99.2 | 3028938 | 1234992 |  | 4.0 |  | Milan |
|  | 4DL | 2.3-23.5 | 39561145 | 1138232 | 7.2 | 10.0 | 6.8 | Milan |
|  | Accumulated percentage of variation explained | | | | 7.2 | 14.0 | 12.6 |  |
| **CM** | 2A | 92.0-96.0 | 984222 | 4993093 | 7.6 | 15.5 | 9.1 | Milan |
|  | 3AS | 15.7-26.5 | 1119071 | 1130676 |  | 10.0 | 8.7 | Caninde#2 |
|  | 5BS | 20.7-23.0 | 1054478 | 1103503 |  | 6.3 |  | Caninde#2 |
|  | 7AS | 40.3-50.5 | 100033324 | 990824 | 7.1 |  |  | Caninde#2 |
|  | 7DS | 0-16.3 | 1089662 | 1012066 |  |  | 4.0 | Caninde#2 |
|  | Accumulated percentage of variation explained | | | | 14.7 | 31.8 | 21.8 |  |

**Fig. S1** Alignment of the 7AL QTL regions in the populations Alondra/Milan (AM) and Caninde#2/Milan-S (CM). DArTSeq markers are indicated on the outer sides of the linkage groups, whereas marker positions are indicated on the inner sides. Markers in the QTL regions are highlighted in red, and those shared between the two populations are connected with dotted lines. Linkage groups are only partially presented to show the QTL regions.
